# A remarkable new species of the sharpshooter genus *Egidemia* (Insecta, Hemiptera, Cicadellidae, Cicadellinae)

**DOI:** 10.3897/zookeys.97.741

**Published:** 2011-05-11

**Authors:** Gabriel Mejdalani, Cláudia Garcia

**Affiliations:** Departamento de Entomologia, Museu Nacional, Universidade Federal do Rio de Janeiro, Quinta da Boa Vista, São Cristóvão, 20940–040, Rio de Janeiro, RJ, Brasil

**Keywords:** Auchenorrhyncha, Colombia, identification key, leafhopper, Proconiini, taxonomy

## Abstract

A new species of *Egidemia* China, 1927, *Egidemia impudica*, is described and illustrated from the Department of Magdalena (Colombia). The male genitalia of the new species have a very peculiar, diagnostic feature: the pygofer is considerably reduced and truncate posteriorly, so that part of the aedeagus is exposed. A key to males of all known *Egidemia* species is provided. Notes comparing *Egidemia impudica* with the other nine known species of the genus are also given.

## Introduction

The sharpshooter genus *Egidemia* China, 1927 currently includes nine species (Carpi and Mejdalani 2010): *Egidemia anceps* (Fowler, 1899), type species, *Egidemia fowleri* (Distant, 1908), *Egidemia gracilis* Schröder, 1972, *Egidemia inflata* Young, 1968, *Egidemia obtusata* (Melichar, 1925), *Egidemia paranceps* Young, 1968, *Egidemia peruana* Carpi & Mejdalani, 2010, *Egidemia proxima* (Melichar, 1925), and *Egidemia speculifera* (Walker, 1851). This genus is widespread in the Neotropical region, being recorded from Mexico and Panama to Peru, Brazil, and Argentina (Young 1968). *Egidemia* was also recorded from Colombia by Freytag and Sharkey (2002). However, it should be noted that the box 2 of Freytag and Sharkey (2002), in which a synopsis of Colombian Cicadellidae is provided, indicates that the genus is not known from Colombia, whereas their taxonomic list mentions the record of *Epidemia* [sic] sp. from the Colombian Department of Magdalena. *Egidemia* can be distinguished from other genera of the Proconiini by the following combination of features (see key of Young 1968): (1) frons with texture of dorsomedian area granular; (2) metameron exposed when the forewings are in rest position; (3) metepimeron with shelflike projection; (4) forewings hyaline or translucent and (5) with the claval veins consistently fused through a considerable portion of their length. The reader is referred to Carpi and Mejdalani (2010) for additional notes on the taxonomy and possible phylogenetic relationships of *Egidemia* to other genera of the Proconiini.

We describe herein a remarkable new *Egidemia* species from Colombia (Department of Magdalena). The description is based on the material that Freytag and Sharkey (2002) employed to provide the above-mentioned record of *Epidemia* [sic] sp. from Colombia. We consider the new species remarkable because its male pygofer is considerably reduced and with a truncate posterior margin, so that part of the aedeagus is exposed, a very peculiar feature for a Proconiini sharpshooter. A new key to males of the species of *Egidemia*, modified from that of Carpi and Mejdalani (2010), is provided.

## Material and methods

Techniques for preparation of the male genital structures follow Oman (1949). The dissected parts are stored in microvials with glycerin and attached below the specimens, as suggested by Young and Beirne (1958). The morphological terminology adopted herein follows mainly Young (1968), except for the facial areas of the head (Hamilton 1981, Mejdalani 1998). Digital images of eight of the nine known *Egidemia* species (body in dorsal and lateral views) are now available in the internet site “Sharpshooter Leafhoppers of the World” (Wilson et al. 2009). These images were useful for the comparisons carried out in the present study. The specimens herein described belong to the Instituto Alexander von Humboldt (IAHC), Villa de Leyva (Colombia) and to the Museu Nacional (MNRJ), Universidade Federal do Rio de Janeiro, Rio de Janeiro (Brazil). Label data are given inside quotation marks with a reversed virgule (\) separating lines on the labels. The photograph of the body in dorsal view was prepared with the software Automontage (Synoptics Inc., Frederick, Maryland, USA) using a digital camera attached to a stereomicroscope.

## Results

**Genus *Egidemia* China, 1927**

### Egidemia impudica sp. n.

urn:lsid:zoobank.org:act:3AB00DB0-9D69-4B29-A19D-D70212713323

http://species-id.net/wiki/Egidemia_impudica

**Figure F2:**
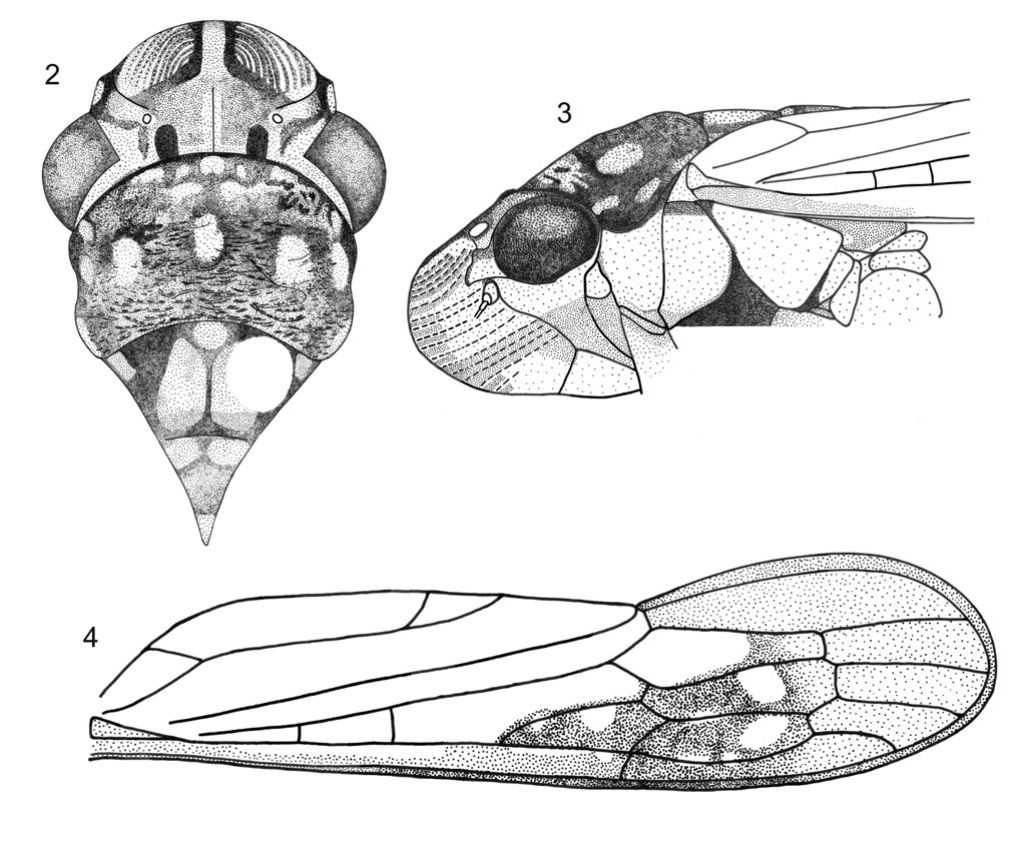
**Figures 2–4.** *Egidemia impudica* sp. n. **2** crown, pronotum and mesonotum, dorsal view (the white circle on the mesonotum is the pin perforation) **3** anterior portion of body, lateral view **4** left forewing.

**Figure F3:**
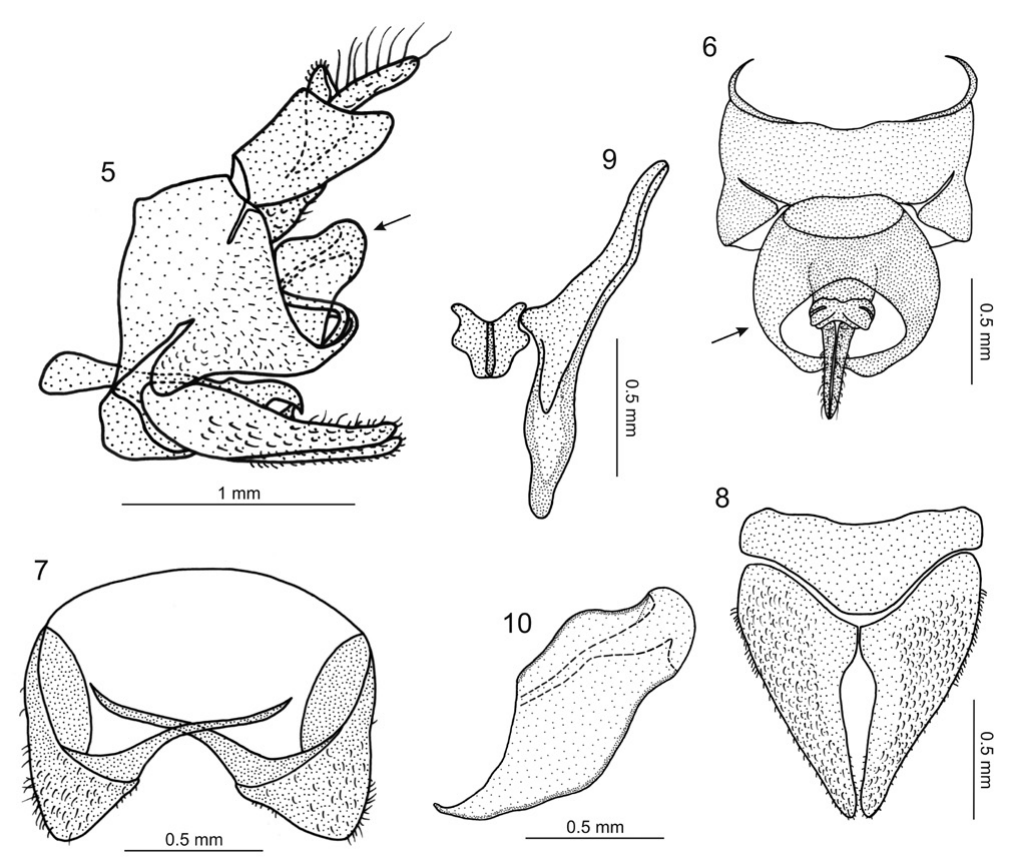
**Figures 5–10.** *Egidemia impudica* sp. n., male genitalia **5** genital capsule, lateral view (arrow indicates the exposed aedeagus) **6** pygofer and anal tube, dorsal view (arrow indicates the expanded segment X of the anal tube) **7** pygofer, caudal view **8** valve and subgenital plates, ventral view **9** connective and right style, dorsal view **10** aedeagus, lateral view.

Figs 1 [Fig F2] [Fig F3] 10

#### Description of the male holotype.

Length, 11.5 mm (male paratype, 12 mm) including wings in repose. Head (Fig. 2), in dorsal view, well produced anteriorly; median length of crown approximately seven-tenths interocular width and four-tenths transocular width. Crown (Fig. 2), in dorsal view, with anterior margin broadly rounded; without carina at transition from crown to face; without median fovea; ocelli located slightly behind imaginary line between anterior angles of eyes, each ocellus closer to adjacent eye angle than to median line of crown; without longitudinal keel laterad of each ocellus; with broad M-shaped elevation bordering posterior margin; with pubescence; frontogenal sutures extending onto crown and approaching ocelli; coronal suture distinct. Antennal ledges (Fig. 2), in dorsal view, protuberant; in lateral view (Fig. 3), with dorsal carina, anterior margin strongly declivous and with concavity. Face (Fig. 3) pubescent, especially on inferior portions; frons convex, swollen, muscle impressions distinct, median portion granulate; epistomal suture incomplete medially; clypeus not produced, its contour continuing profile of frons.

**Figure:**
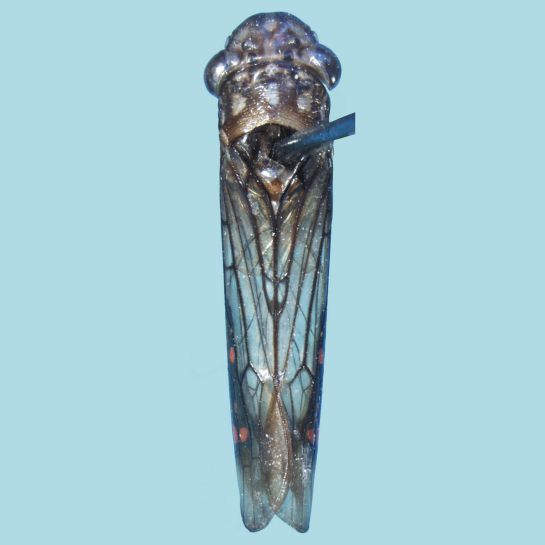
**Figure 1.** *Egidemia impudica* sp. n. Male holotype (IAHC), body in dorsal view (antennae and legs not depicted, abdomen removed for dissection). Length, 11.5 mm.

Thorax (Fig. 2), in dorsal view, with pronotal width less than transocular width of head; pronotum with lateral margins slightly sinuous and slightly divergent anteriorly; pronotal surface rugose and punctate (except on anterior third) and pubescent; posterior margin distinctly concave; dorsopleural carinae (Fig. 3) complete, slightly arched downward anteriorly, strongly declivous posteriorly. Mesonotum (Fig. 2) with scutellum only very slightly striate. Forewings (Fig. 4) mostly hyaline with large sclerotized area extending mainly over outer discal cell, outer and median anteapical cells and adjacent portions of costal margin; veins elevated and distinct; claval veins fused through most of their length, separated only basally and apically; outer discal cell reduced, about half length of inner discal cell; with three closed anteapical cells (inner one broadened anteriorly) and four apical cells, base of fourth more proximal than base of third; without anteapical plexus of veins and without supernumerary anteapical cross veins to costal margin. Hindwings extending almost as far posteriorly as forewings; vein R2+3 incomplete. Hindleg with femoral setal formula (visible only on right leg of holotype) 2:1:1:1 (with additional, unaligned slender seta located anteriorly to the row of three setae; this additional seta absent in the male paratype); length of first tarsomere less than combined length of second and third ones; first tarsomere with two parallel rows of small setae on plantar surface.

Color. Anterior dorsum (Figs 1–3) mostly brown. Crown with three maculae anteriorly (median one elongate), outer portion of antennal ledges, macula adjacent to inner eye margin, area around ocelli, and elongate macula from posterior margin to interocellar portion, pale yellow; inner portion of antennal ledges and pair of conspicuous maculae on posterior coronal margin, dark brown. Pronotum with irregular maculae on anterior third, five distinct, transversely aligned maculae on median third and pair of maculae on posterior third at lateral margins, pale yellow. Mesoscutum with median macula basally, pair of maculae basilaterally, and pair of irregular areas medially, pale yellow; mesoscutellum with pair of maculae basally and macula on apical portion, pale yellow. Forewings (Figs 1, 4) mostly translucent with brown veins; small brown area along basal portion of costal margin; distal half of costal margin, outer discal cell, outer anteapical cell, median anteapical cell, and part of inner anteapical cell brown (mostly darker than other wing portions); outer discal, outer anteapical, and median anteapical cell each with distinct orange macula; additional orange macula on costal area adjacent to anterior limit of outer anteapical cell; additional irregular yellow to orange marks also present in this area; apical cells brown. Body (Fig. 3), in lateral view, with broad yellow area extending from lateral portions of frons to posterior limit of thorax, bordered inferiorly by irregular brown marks. Face (Fig. 3) mostly pale yellow; muscle impressions and diffuse area on median portion of frons, brown to dark brown.

Male genitalia with pygofer (Fig. 5), in lateral view, short, considerably reduced posteriorly, exposing aedeagal shaft; posterior margin obliquely truncate; ventroapical portion with conspicuous long process directed mesally; in caudal view (Fig. 7), processes crossing each other medially; pygofer surface with small setae distributed mostly ventrally and on posterior half. Valve (Fig. 8), in ventral view, with short lateral margins; posterior margin distinctly produced posteriorly. Subgenital plates (Fig. 8), in ventral view, triangular, narrowing gradually toward apex; not fused to each other, close to each other for short distance on basal portion and then with distinct space between inner margins; surface with many scattered small setae; in lateral view (Fig. 5), plates extending beyond pygofer apex, with small dentiform projection associated with style apical portion. Connective (Fig. 9), in dorsal view, broadly Y-shaped with both arms and stalk short; with short median keel. Styles (Fig. 9), in dorsal view, elongate, extending posteriorly distinctly beyond apex of connective, portion before connective approximately of same size as portion behind it; apical portion directed posteriorly, not distinctly curved; apex obtuse. Aedeagus (Fig. 10) symmetrical; shaft, in lateral view, simple, directed dorsally, lobulate apically; dorsal and ventral margins sinuous; gonopore located on apex. Paraphyses absent. Anal tube (Figs 5, 6), in dorsal view, strongly developed in comparison to pygofer size; segment X (Fig. 5), in lateral view, longer than dorsal pygofer margin, expanded toward apex; in dorsal view (Fig. 6), broad, distinctly rounded.

#### Female

unknown.

#### Type specimens.

Colombia, Magdalena Department. Male holotype (IAHC) with labels “COLOMBIA Magdalena \ PNN Tayrona Zaino \ 11º20’N 74º2’W 50 m” and “Malaise 7/17/00-7/28/00 \ R. Henriquez, leg. M.299”. Male paratype (MNRJ) with same data as holotype, excepting “6/14/00-6/29/00” and “M.240”.

#### Etymology.

The new species name, *impudica*, refers to the distinctly reduced male pygofer, which results in the partial exposure of the aedeagus.

## Remarks

Considering the known species of *Egidemia*, the new taxon appears to be most similar to *Egidemia inflata*, both in the color pattern (especially the maculae of the pronotum and forewings) and in certain aspects of the male genitalia (aedeagus, styles, and inner margin of the subgenital plates). *Egidemia inflata* is recorded from Mexico and Belize (Young 1968, McKamey 2007), whereas the new species is known only from Colombia. *Egidemia impudica* can be easily distinguished from *Egidemia inflata*, as well as from the remaining species of the genus, by the following features: (1) male pygofer reduced with (2) obliquely truncate posterior margin (Fig. 5) and (3) a pair of very elongate processes on ventroapical area that cross each other medially (Fig. 7); (4) subgenital plates extending beyond pygofer apex (Fig. 5). Due to the presence of the first two features, the aedeagus is partially exposed. To provide a comparison with *Egidemia impudica*, we have redrawn the illustrations of Young (1968) of the pygofer (Fig. 11), pygofer process (Fig. 12) and aedeagus (Fig. 13) of *Egidemia inflata*. We have added the new species to the key of Carpi and Mejdalani (2010) to males of *Egidemia*. The new key also mentions the countries from which each species has been recorded (based on Young 1968, McKamey 2007, Takiya and Dmitriev 2007 and Carpi and Mejdalani 2010).

**Figure:**
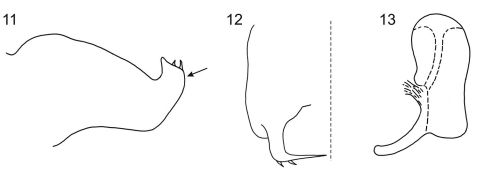
**Figures 11–13.** *Egidemia inflata* Young, 1968 **11** pygofer, lateral view (arrow indicates the process) **12** apical portion of pygofer, caudoventral view **13** aedeagus, lateral view. These figures, redrawn from Young (1968), are in the public domain.

## Key to males of *Egidemia* (modified from Carpi and Mejdalani (2010) to include *Egidemia impudica* sp. n.)

**Table:** 

1	Aedeagus with processes	2
–	Aedeagus without processes	6
2	Aedeagus with symmetrical processes	3
–	Aedeagus with asymmetrical processes (Young 1968: Fig. 169g)	*Egidemia proxima* (Melichar, 1925) (Mexico)
3	Aedeagus, in lateral view, with distinct curved lobe arising dorsoapically above pair of strong spiniform processes (Schröder 1972: Fig. 1b)	*Egidemia gracilis* Schröder, 1972 (“Amaz.” [Amazon region])
–	Aedeagus, in lateral view, without such dorsoapical lobe	4
4	Pygofer processes arising dorsoapically (Young 1968: Fig. 164c)	5
–	Pygofer processes arising ventrally; pygofer, in lateral view, curved dorsally and with truncate apex (Young 1968: Fig. 170c)	*Egidemia obtusata* (Melichar, 1925) (Peru)
5	Styles, in dorsal view, slightly expanded apically; aedeagal processes, in caudoventral view, very short, their length not more than four times their width (Young 1968: Fig. 165g)	*Egidemia paranceps* Young, 1968 (Costa Rica, Nicaragua, Panama)
–	Styles, in dorsal view, not expanded apically; aedeagal processes, in caudoventral view, with length many times their greatest width (Young 1968: Fig. 164g)	*Egidemia anceps* (Fowler, 1899) (Mexico, Guatemala, Panama)
6	Pygofer, in lateral view, short, partially exposing aedeagus (Fig. 5), ventroapical margins with pair of elongate processes that cross each other medially (Figs 5, 7)	*Egidemia impudica* sp. n. (Colombia)
–	Pygofer, in lateral view, elongate, not exposing aedeagus, ventroapical margins without pair of elongate processes that cross each other medially	7
7	Aedeagus, in lateral view, with shaft rectilinear (Carpi and Mejdalani 2010: Fig. 8); styles, in dorsal view, with apical portion directed outward (Carpi and Mejdalani 2010: Fig. 7)	*Egidemia peruana* Carpi and Mejdalani, 2010 (Peru)
–	Aedeagus, in lateral view, with shaft curved dorsally (Young 1968: Fig. 167f); styles, in dorsal view, with apical portion directed posteriorly (Young 1968: Fig. 168e)	8
8	Pygofer process branched (Young 1968: Fig. 167c)	*Egidemia fowleri* (Distant, 1908) (Mexico)
–	Pygofer process not branched (but may bear small teeth)	9
9	Aedeagus, in lateral view, narrowest in apical half of its length (Young 1968: Fig. 169f*); posterior pygofer margin, in lateral view, narrowly round (Young 1968: Fig. 168c)	*Egidemia speculifera* (Walker, 1851) (Brazil, Paraguay, Argentina)
–	Aedeagus, in lateral view, inflated, broadest in apical half of its length (Fig. 13); posterior pygofer margin, in lateral view, forming broad process directed dorsally (Fig. 11)	*Egidemia inflata* Young, 1968 (Mexico, Belize, Cuba [?])

* Note. There is a mistake in the numbers of *Egidemia* figures in Young’s (1968) paper. The aedeagi of *Egidemia speculifera* and *Egidemia proxima* had their numbers exchanged. Figure 168f is actually *Egidemia proxima*, instead of *Egidemia speculifera* as given in his legend, whereas figure 169f is *Egidemia speculifera* (*Egidemia proxima* in the legend).

## Supplementary Material

XML Treatment for Egidemia impudica
